# Efficacy of Shenqi Pollen Capsules for High-Altitude Deacclimatization Syndrome via Suppression of the Reoxygenation Injury and Inflammatory Response

**DOI:** 10.1155/2019/4521231

**Published:** 2019-11-15

**Authors:** Binfeng He, Mingdong Hu, Zhihui Liang, Qianli Ma, Yunhai Zi, Zhiwei Dong, Qi Li, Yongjun Luo, Guisheng Qian, Liang Guo, Kexiong Lin, Zhenyu Liu, Guansong Wang

**Affiliations:** ^1^Institute of Respiratory Diseases, Xinqiao Hospital of the Third Military Medical University, Chongqing 400037, China; ^2^Bethune International Peace Hospital of PLA, Shijiazhuang, Hebei 050000, China; ^3^Center for Disease Control and Prevention of Zhengzhou City and First Affiliated Hospital of Zhengzhou University, Zhengzhou, Henan 450000, China; ^4^College of High Altitude Military Medicine, Third Military Medical University, Chongqing 400038, China; ^5^Department of Respiration, Tongren City People's Hospital, Guizhou 554300, China; ^6^Department of Emergency, The First Affiliated Hospital of Nanchang University, Nanchang 330006, China

## Abstract

High-altitude deacclimatization syndrome (HADAS) is involved in hypoxia-reoxygenation injury and inflammatory response, induced a series of symptoms, and has emerged as a severe public health issue. Here, we investigated the mechanism as well as potential means to prevent HADAS using Shenqi pollen capsules (SPCs) in subjects with HADAS in a multicenter, double-blinded, randomized, placebo-controlled study. All subjects were at the same high altitude (3650 m) for 4-8 months before returning to lower altitudes. Subjects (*n* = 288) in 20 clusters were diagnosed with mild or moderate HADAS on the third day of the study. We randomly allocated 20 clusters of subjects (1 : 1) to receive SPCs or a placebo for 7 weeks, and they were then followed up to the 14^th^ week. The primary endpoints were subjects' HADAS scores recorded during the 14 weeks of follow-up. Compared with the placebo, SPC treatment significantly decreased the subjects' HADAS scores and reduced the incidence of symptom persistence. SPC therapy also reduced the serum levels of CK, CK-MB, LDH, IL-17A, TNF-*α*, and miR-155 and elevated IL-10 and miR-21 levels. We thus demonstrate that SPCs effectively ameliorated HADAS symptoms in these subjects via suppression of the hypoxia-reoxygenation injury and inflammatory response.

## 1. Introduction

High-altitude deacclimatization syndrome (HADAS) is a serious form of high-altitude deacclimatization (HADA), in which the loss of high-altitude acclimatization provokes complex and multifaceted physiological and functional changes in individuals who have acclimated to a high altitude and then returned to a lower altitude or sea level [1–7]. In general, HADA occurs under typical environmental reacclimatization in which individuals return to lower altitudes from high altitudes. Similar to the process of high-altitude acclimatization (HAC), the physiological processes associated with the progression of HADA in a time-dependent manner: acclimatory changes and acquired hypoxia tolerance are not lost immediately upon returning to lower altitudes but instead disappear gradually over time. HADA also involves several physiological adjustments, including decreased erythrocyte, hemoglobin, and hormone levels [8]. Interestingly, HADA symptoms are similar to those of individuals who suffer from high-altitude stress. A previous study showed that migrants from high plateaus suffered from excessive somnolence, diminished reflexes, and inadequate fine motor coordination while living at sea level [9]. Cui and colleagues showed that 70.76% of individuals who live at high altitudes for 10–30 years suffered from a series of clinical symptoms, including fatigue, headaches, and sleepiness while undergoing HADA [10]. Individuals undergoing HADA can suffer from multiple symptoms, including sleepiness, insomnia, unresponsiveness, memory loss, fidgetiness, headache, throat pain or discomfort, coughing, expectoration, chest tightness, flustering, increased appetite or decreased appetite, diarrhea, abdominal distention, abdominal pain, lumbago, and arthralgia [1, 11]. In addition, cardiovascular, hematological, and respiratory system abnormalities were observed in subjects when they returned to lower altitudes [7, 11]. Other studies have shown that several of these symptoms can last many years in some severe cases, and 1‰ of subjects experienced such severe symptoms that they had to return to high altitudes [12]. We refer to all of the above pathological conditions as features of HADAS [1, 13, 14].

HADAS is an important public health issue. First, there are a large number of individuals with HADAS owing to the increasing number of individuals who have worked and traveled to high altitudes worldwide. In China, more than one million people live and work at high altitudes. They return to lower altitudes when they have finished their work, for trade contacts or other reasons. Some of them will develop HADAS [8, 15]. Cui demonstrated that 71% of individuals who lived at high altitudes for 10 to 30 years suffered from HADAS upon returning to lower altitudes [10]. Our previous study showed that 84% of individuals who lived in Tibet for 10 to 20 years had suffered from HADAS [11]. Additionally, the incidence of HADAS was very high, reaching 100%, in individuals who worked in earthquake relief at Yushu in Qinghai Province [15]. Second, HADAS severely impairs the quality of life of affected individuals. Previous studies showed that subjects with HADAS suffer a series of clinical symptoms, including sleepiness, insomnia, unresponsiveness, memory loss, and fidgetiness [1, 10, 11]. Another study showed that some subjects who had worked at high altitudes for 20 years (Tibet and Qinghai Province, >3000 m) suffered from severe HADAS, with symptoms lasting many years [12]. Although HADAS has received increasing attention in recent years from the medical community, treatment options are limited and are often ignored by public health advocates.

Treating HADAS remains a major challenge. Recently, several studies showed some traditional Chinese drugs and hyperbaric oxygen (HBO) treatments could relieve symptoms of subjects with HADAS. Shenqi pollen capsules (SPCs) consist of a compound prepared with the following raw materials [16]: *Codonopsis pilosula*, the root *of Astragalus propinquus*, cattail pollen, zasiokaurin, and sunflower pollen. SPCs have been processed and analyzed by colorimetric methods and high-performance liquid chromatography. The active ingredients of SPCs include flavonoids, quercetin, kaempferol, isorhamnetin, astragaloside, atractylenolide III, and lobetyolin. Our previous study showed that SPCs, Herba Rhodiolae capsules, and Sankang capsules were effective in improving the symptoms of HADAS compared with the effects of a placebo, and SPCs were better at decreasing HADAS scores compared to the effects of Rhodiolae capsules and Sankang capsules in a single-blinded, randomized, controlled trial [17]. Another study examined 380 subjects (280 males and 100 females) who had worked building a factory at high altitude (4300 m) for 5 days and then returned to camp (2800 m) for 2 days. Over the 3 years of the study, 82.37% of the subjects felt uncomfortable or suffered from HADAS symptoms, including sleepiness, insomnia, and unresponsiveness. The symptoms were partly alleviated in 117 subjects who took SPCs orally for 2 weeks [18]. In addition, Cui et al. showed that *Ginkgo biloba* leaf extract tablets, compound *Codonopsis pilosula extract* capsules, and Herba Rhodiolae capsules decreased plasma viscosity and improved tissue microcirculation in subjects with HADAS compared to the effects of a placebo [19]. Jin and colleagues demonstrated that dizziness, chest tightness, and memory loss was significantly relieved after subjects with HADAS underwent 5 rounds of HBO therapy [20].

Although previous study showed SPCs can be effective in treating HADAS in short-term, single center trials, these trials did not fully clarify the long-term effectiveness and safety of SPCs in treating HADAS. Therefore, we aimed to evaluate the efficacy of SPCs for treating HADAS and explore its novel mechanism.

## 2. Methods

### 2.1. Study Design

A multicenter, double-blinded, placebo-controlled, randomized trial was performed. All subjects presented at four centers in four hospitals: Xinqiao Hospital (in Chongqing), the 478^th^ Hospital of PLA (in Kunming), First Hospital of Zhengzhou University (in Zhengzhou), and Wuwei City People's Hospital (in Wuwei). The trial methods were carried out in accordance with the approved guidelines. All subjects provided written informed consent before enrollment. The researchers conducting this trial were respiratory and ultrasonography physicians at the four hospitals. The screening, enrollment, and study visits occurred at the four trial sites, and the safety of the study, as well as the data, was monitored by an independent data and safety monitoring board.

All researchers were trained in the assessment of HADAS symptoms, including the classification decisions and scoring criteria, in group sessions at four meetings throughout the trial and in individual training sessions with the trial manager. All researchers recruited between meetings were trained by the trial manager, and local supplementary training was provided whenever necessary. This study was approved by the medical ethics committee of Xinqiao Hospital and the Third Military Medical University, and all subjects provided written informed consent. The report of the study adheres to the Consolidated Standards of Reporting Trials (CONSORT) statement (Supplementary [Supplementary-material supplementary-material-1]), and the trial was registered at Chinese Clinical Trial Register (ChiCTR) with the identifier number ChiCTR-TRC-12002653 (URL: http://www.chictr.org.cn/showproj.aspx?proj=6899).

### 2.2. Subjects

Enrollment occurred from February 2011 through October 2012. Individuals aged 18–60 years were eligible for inclusion if they were diagnosed with HADAS and presented scores of 6 to 25 upon returning to a lower altitude from Lhasa (3650 m), where they had worked for 4–7 months [1]. The subjects were followed up with active surveillance. The principal exclusion criteria comprised symptoms directly attributable to primary diseases affecting the cardiovascular, respiratory, nervous, urinary, and hematological systems; cancer or leukemia; and a recent history of influenza, upper respiratory tract infection, infectious diarrhea, or similar symptoms.

The subjects from the cities of Chongqing (180 m), Kunming (1800 m), Zhengzhou (110 m), and Wuwei (1500 m) worked at four spring water or beer factories at the same altitude (3650 m). The working conditions and intensity and the dietary patterns of the subjects were similar to those of individuals at the lower altitude factories where the subjects originally worked.

### 2.3. Randomization and Masking

In our study, 10 to 17 subjects always lived and worked together as a cluster, for a total of 20 units (*n* = 288) across the research centers. Because the subjects who lived in a given unit might exchange drugs under the masked conditions, we considered each unit as one cluster when applying random assignments, i.e., all subjects in a unit took the same drug. The 20 clusters were randomly assigned (1 : 1) to receive SPCs or a placebo. The randomization sequence was computer generated using permuted block randomization with a block size of four by an individual who was independent of the study team. The allocation was concealed using vials coded for each subject. At every drug-refill visit, a constructor assigned the container numbers to be dispensed to the subjects. Henan Fenghuang Drugs Manufacture Stock Co. Ltd. (Xinxiang City, Henan Province) prepared the trial drugs and containers, which were labeled with numbers according to the randomized treatments to ensure that the stocks matched the assignments at each site.

All subjects, physicians, outcome assessors, inspectors, and researchers (persons conducting the study) were blinded to the treatment allocations until the study was unblinded and completed, and the database was frozen. The vials for SPCs and placebo capsules and their contents were indistinguishable in external appearance, texture, taste, and smell.

### 2.4. SPC Production

SPCs were manufactured with raw material according to a previous patent [16] in Henan Fenghuang Drugs Manufacture Stock Co. Ltd. (Xinxiang City, Henan Province). Briefly, 4 kg *Codonopsis pilosula* and 4 kg Mongolian milkvetch root were mixed, and their active ingredients were extracted by water extraction. These active ingredients were concentrated, dried, and made into powdered extracts, which then underwent size reduction. Additionally, 1 kg each of corn pollen, sunflower pollen, rape bee pollen, cattail pollen and honey was mixed and fermented at 37°C for 72 h. The fermentation products were dried, made into a powder, and mixed with the powdered *Codonopsis pilosula* and Mongolian milkvetch extract. The mixed products were then made into Shenqi pollen capsules (SPCs).

### 2.5. Procedures

The subjects ingested three SPCs or placebo capsules orally three times a day (t.i.d.) for 7 weeks. The subjects also underwent follow-up visits at 7 and 14 weeks.

The study procedures included checking vital signs upon returning to a lower altitude from a high altitude; the subjects' level of discomfort and symptoms were evaluated and recorded on days 1 through 7. The symptom scores, routine blood analyses, myocardial enzyme levels, and heart function assessments were evaluated on the third day after the subjects returned to a lower altitude from a high altitude, similar to our previous study. The subjects were evaluated at the four sites at 7 and 14 weeks after the administration of the drug or a placebo. Subjects were enrolled in our study upon being diagnosed with mild or moderate HADAS [1, 3], and they began to take SPCs or placebo capsules on the fourth day after returning to a lower altitude from a high altitude. All subjects were followed up by telephone or oral interview each month until the end of the trial. If any other medication or therapy was required to treat concomitant diseases, the name of the drug or therapy, actual dosage, dosing frequency, and start/stop time were well-documented. Adverse events (AEs) reported by the subjects or observed by the investigator were recorded regardless of whether they were considered to be drug related and were reported according to the State Food and Drug Administration Criteria.

### 2.6. Diagnostic and Scoring Criteria for HADAS

Establishing diagnostic and scoring criteria is crucial to further explore the pathophysiology of HADAS, as well as its prevention and treatment. The diagnostic criteria for HADAS were first reported in Zhang et al.'s book *People and High Altitudes* [13] in 1996. Based on a large-scale epidemiological investigation, we modified these diagnostic criteria and established a scoring criterion to more accurately evaluate HADAS and explore its pathophysiology [1, 11]. This system contains essential diagnostic criteria, auxiliary diagnostic criteria, and exclusion criteria for HADAS, as well as a classification and scoring criteria of the symptoms of HADAS. Symptom scores were evaluated according to the scoring criteria for fixed-duration high-altitude deacclimatization syndrome (the details in Supplementary [Supplementary-material supplementary-material-1]).

### 2.7. Outcome Measures

The primary outcome was the subjects' HADAS scores in the records taken over the 14 weeks. The HADAS scoring criteria had been previously validated in adults living at high altitudes and who were acclimatized to high-altitude environments before returning to a lower altitude. The scores ranged from 0 to 63 (0 to 5 points representing no HADAS symptoms and 26 or more points representing very severe symptoms). The scores were evaluated by a physician who had received specialized training.

The secondary endpoints included the levels of routine blood measures (RBC, Hb, and Hct levels), myocardial enzyme (creatine kinase (CK), CK-MB, and LDH), and measures of heart function (e.g., LVEF) in the subjects with HADAS. Routine blood and myocardial enzyme analyses were performed as previously described at the 7- and 14-week follow-up visit as previously described [1]. Serum was isolated and stored at -80°C until the assays were conducted. A color Doppler ultrasonography system (GE LOGIQ-3) was used to measure the LVEF and LVFS and other parameters. The heart function of all subjects was evaluated by the ultrasonography specialist from Xinqiao Hospital (the details in Supplementary [Supplementary-material supplementary-material-1]).

### 2.8. Evaluation of the Level of Serums IL-17A, IL-10, and TNF-*α*

The ELISA kit for human serum IL-17A, TNF-𝛼, and IL-10 ELISA kits was ordered from R&D Systems (Abingdon, UK). The level of IL-17A, TNF-𝛼, and IL-10 levels was detected according to the manufacturer's instructions [21].

### 2.9. Detection of the Levels of Serums SOD and MDA

The analysis kit of serums SOD and MDA was ordered from the Nanjing Jiancheng Bioengineering Institute (Nanjing, China). All samples had been evaluated according to the previous study [21].

### 2.10. Isolation of Serum Small RNA and Analysis of the Level of miR-155 and miR-21

Total RNA was isolated from subject's serum samples according to a previous study [22]. Briefly, these serum samples were pretreated with proteinase K in 37°C for 30 min, and synthetic C. elegans microRNA 39 (cel-miR-39), as an external reference, was added into these samples. Small RNA was isolated using TRIzol reagent (Sigma, Lot: #T9242) according to the manufacturer's instructions. Small RNAs were reverse-transcribed using miRNA First Strand cDNA Synthesis (Tailing Reaction) (Sangon Biotech, Lot: # B532451), and qPCR were carried out with the following conditions: 95°C for 10 min, 95°C for 25 s, and 60°C for 30 s at the annealing temperature through 40 cycles. The data was analyzed as previous description [23].

### 2.11. Statistical Analysis

We calculated that a sample size of 288 individuals was needed to provide 80% power to detect a two-point difference in HADAS scores between the SPC and the placebo groups at 7 and 14 weeks, with a type I error rate of 0.05 and allowing for a 20% loss during follow-up. An interim analysis using the O'Brien-Fleming stopping boundaries was performed after 173 subjects were recruited. All analyses were based on intention-to-treat (ITT) analyses, defined as an analysis of all randomized individuals who received at least one dose of the study drug. Missing data were handled using the last observation carried forward (LOCF) imputation technique. All subjects in the random assignment of the 20 clusters were included in the final analyses. Missing values were assumed to be missing at random. Two-sided tests with *p* values less than 0.05 were considered statistically significant.

We summarized continuous variables as the means (SE, 95% CIs) and categorical variables as *n* (%). Statistical differences between groups were analyzed using a *t* test for quantitative data and Fisher's exact test for categorical data at baseline. The analysis of the primary endpoint, the treatment-related difference in the mean difference from baseline to measurements taken at follow-up, used a linear mixed-effects model for repeated measures and represents the average treatment effect at weeks 7 and 14. The model included main effects for time, treatment group, height differences, work intensity, smoking status, alcohol consumption status, and occurrence of acute mountain sickness (AMS) at high altitude and age, as well as the interaction between the time and the treatment group as fixed effects, whereas subject differences were included as random effects with an unstructured within-subject variance-covariance structure, and results were estimated using a restricted maximum likelihood model. Continuous variables for the secondary outcomes were evaluated using the same linear mixed-effects model for repeated measures used in the analysis of the primary endpoint. In cases of interactions, the effects of a group at each time point were compared, and the Bonferroni corrections were used to adjust for multiple comparisons.

The incidence of symptoms and HADAS severity were compared between the SPC and the placebo groups using Fisher's exact test. The overall incidence of adverse events and symptoms of drug-related events in both groups were analyzed by Fisher's exact test. SPSS 15.0 for Windows (SPSS Inc., Chicago) was used for all statistical analyses.

## 3. Results

**Figure 1:**
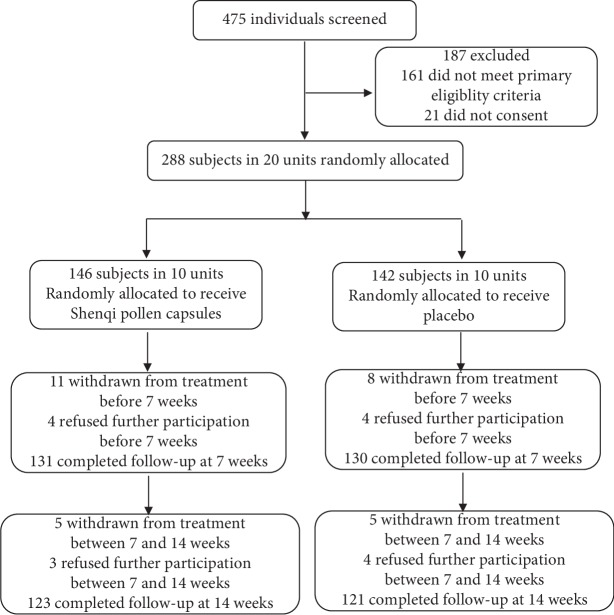
The flow diagram of the trial.

The flow diagram for subject selection and follow-up in the trial is shown in Figure 1. We recruited subjects between January 2011 and March 2012, and follow-up interviews were completed by June 2012. A total of 475 subjects were assessed for eligibility, of whom 288 were enrolled. Subjects were randomly assigned to two groups: 146 (50.69%) received SPC treatment and 142 (49.31%) received placebo treatment.

Of the 146 individuals in the SPC group, 23 (15.75%) had withdrawn by week 14, as had 21 (14.78%) of the 142 subjects in the placebo group. By week 7, 15 of the 146 individuals in the SPC group had withdrawn (10.27%, 11 withdrew from treatment and 4 refused further participation), as had 12 of 142 from the placebo group (8.45%, 11 withdrew from treatment and 4 refused further participation). Between weeks 7 and 14, 8 of the 131 subjects from the SPC group had withdrawn (6.10%, 5 withdrew from treatment and 3 refused further participation), as had 9 of 130 subjects from the placebo group (5 withdrew from treatment and 4 refused further participation). The dropout rate was not different between the SPC and the placebo groups (*p* > 0.05).

Descriptive, baseline characteristics of the study population are shown in Table 1. No significant differences were found between the SPC and the placebo groups in demographic characteristics, working intensity, smoking status, alcohol consumption status, or incidence of acute mountain sickness (*p* > 0.05).

Primary outcomes at baseline, week 7, and week 14 can be found in Table 2. During the 14 weeks of the study, the subjects' HADAS scores were evaluated by interview or telephone follow-up. The scores decreased from 13.08 to 1.36 in the SPC group and from 12.80 to 2.80 in the placebo group. No significant difference is found between the SPC and the placebo groups for HADAS scores at baseline (*p* > 0.05). There were significant main effects for time, group, and time-group interaction for HADAS scores (*p* < 0.001 for all effects). Mean differences between the groups were -1.34 (95% CI, -1.74 to -0.95; *p* < 0.001) at week 7 and -1.47 (95% CI, -1.87 to -1.07; *p* < 0.001) at week 14. These data indicated that the primary outcomes were dependent on the treatment received, time, and time-group interaction.

The incidence of HADAS symptoms is shown in Table 3. No significant difference was found between the SPC and the placebo groups for symptom incidence at baseline (*p* > 0.05). After 7 weeks of treatment with SPCs or placebo capsules, the incidences of all symptoms decreased over time in both groups. The incidences of symptoms such as sleepiness, insomnia, unresponsiveness, memory loss, agitation, headache, dizziness, coughing, chest tightness, and flustering among subjects in the SPC group were significantly lower at week 7 than they were in the placebo group (*p* < 0.05). The incidences of symptoms such as sleepiness, insomnia, unresponsiveness, headache, dizziness, coughing, expectoration, chest tightness, flustering, and diarrhea were also significantly lower in the SPC group than in the placebo group at week 14 (*p* < 0.05) (Table 3). Additionally, at week 14, the overall proportion of mild reactions was 0.68% in the SPC group, which was significantly lower than the proportion observed in the placebo group (9.15%, *p* < 0.01) (Table 4). Other symptoms did not appear in either group, and no subjects suffered from moderate HADAS at week 14. These data suggest that SPC therapy may ameliorate HADAS symptoms.

Similar to the primary outcome findings, there were significant main effects for time, group, and time-group interaction for creatine kinase (CK), CK-MB, and lactate dehydrogenase (LDH) (*p* < 0.001 for all effects) (Table 5). Additionally, there were significant main effects for time and time-group interaction for pulmonary artery systolic pressure (PASP) (*p*_*t*_ < 0.001 and *p*_*t*+*g*_ < 0.001). The mean differences between the groups in PASP were not significant at week 7 (*p* > 0.05) but were significant at week 14 (*p* < 0.05). Additionally, there were no significant time-group interactions or main effects of group for the secondary outcomes of heart rate (HR), left ventricular ejection fraction (LVEF), left ventricular fractional shortening (LVFS), pulmonary artery inner diameter, or pulmonary artery opening velocity (PAOV) (*p*_*g*_ > 0.05 and *p*_*g*+*t*_ > 0.05) (Table 5). There were significant main effects for time for these parameters (*p*_*t*_ < 0.05), indicating that they are not dependent on the treatment received, as both groups showed similar improvements at each follow-up.

**Figure 2:**
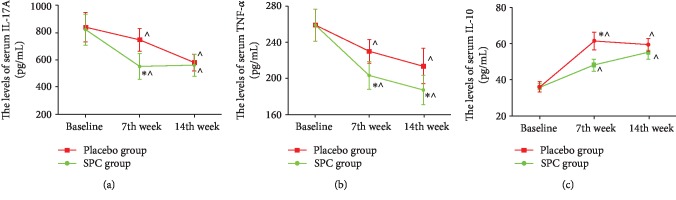
The serum IL-17A, IL-10, and TNF-*α* level of subjects between the SPC and the placebo groups. Data are presented as mean ± SD. The serums IL-17A (a), TNF-*α* (b), and IL-10 (c) of subjects in both groups were assayed at baseline, 7^th^ week, and 14^th^ week. ^∗^*p* < 0.05, relative to the placebo group, ^∧^*p* < 0.05, relative to baseline.

In our previous study, the levels of inflammatory mediators, such as IL-17A and TNF-𝛼, were elevated and the IL-10 level descended upon the subjects returned to sea level from a high-altitude region and were diagnosed as HADAS. Herein, the level of serums IL-17A, IL-10, and TNF-𝛼 was evaluated and compared between the SPC and the placebo groups (Figure 2). After subjects received SPC treatment, the serum concentration of IL-17A and TNF-𝛼 was significantly decreased and the IL-10 level increased at 7^th^ week, compared to the placebo group (*p* < 0.05). At the 14^th^ week, the serum concentration of TNF-𝛼 was lower in the SPC group than in the placebo group (*p* < 0.05). Moreover, the level of IL-17A and IL-10 was no difference between the SPC and the placebo groups (*p* > 0.05).

**Figure 3:**
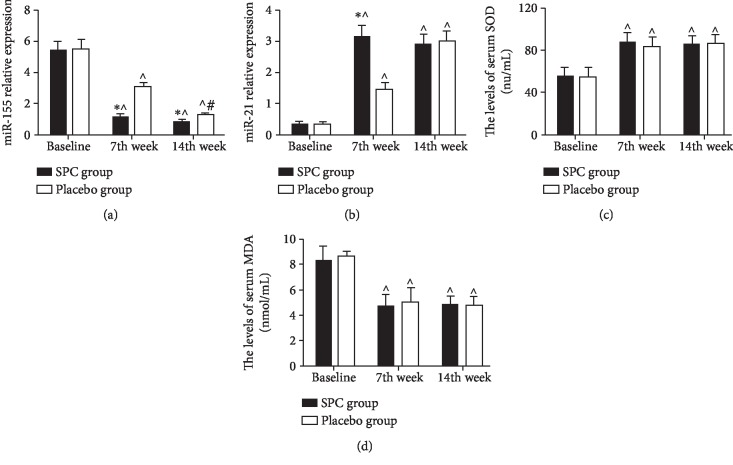
The serum miR-155 and miR-21 level of subjects between the SPC and the placebo groups. Data are presented as mean ± SD. The serums miR-155 (a), miR-21 (b), SOD (c), and MDA (d) of subjects in both groups were assayed at baseline, 50^th^ day, and 100^th^ day. ^∗^*p* < 0.05, relative to the placebo group, ^∧^*p* < 0.05, relative to baseline, and ^#^*p* < 0.05, relative to the 7^th^ week.

miRNAs are small endogenous RNA molecules and relatively stable at serum/plasma. Several reports showed that miRNAs were considered as a biomarker for several diseases [24]. In our study, we found that the serum miR-155 levels of all subjects were notably decreased as time goes on in sea level. After subjects received SPC treatment, the levels of miR-155 were decreased compared to placebo treatment at 50^th^ day and 100^th^ day (*p* < 0.05) (Figure 3(a)). On the contrary, the levels of serum miR-21 of all subjects were elevated at 7^th^ and 14^th^ week, compared to baseline (*p* < 0.05) (Figure 3(b)). Moreover, serum miR-21 levels were higher in the SPC group than the placebo group at 7^th^ week (*p* < 0.05), and no difference between the SPC and the placebo groups (*p* > 0.05). Furthermore, we also analyzed the SOD and MDA level in all subjects' serum. These data showed that there was no difference between the SPC and the placebo groups at 7^th^ week and 14^th^ week (*p* > 0.05).

## 4. Discussion

Exploring the pathological mechanism and recovery process of HADAS is very important. In recent decades, an increasing number of people (more than 1,000,000) who work in commerce, the construction of manufacturing facilities, research and wildlife protection, or for relief agencies have traveled to Tibet and other high-altitude regions (average altitude > 3000 m) in Qinghai or Xinjiang Province in China. Each year, many of these individuals, who are also called temporary migrants, work for 7–10 months at high altitude and then rest for 2 to 3 months at a lower altitude [25]. Our previous study showed that the HADAS symptoms of subjects who were exposed to high altitude for a fixed duration (6 to 8 months) could persist for more than 100 days [1]. HADAS clearly threatens the quality of life of temporary migrants and has become a public health issue. Therefore, it is necessary for physicians and researchers to explore new treatment approaches for HADAS.

To our knowledge, this is the first double-blinded, randomized, controlled study to investigate the potential benefits of traditional Chinese medicine (SPCs) to treat HADAS. Our findings revealed that oral administration of SPCs to subjects with mild or moderate HADAS significantly decreased HADAS scores and symptom incidence in the SPC group compared with the placebo group. Serum CK, CK-MB, and LDH concentrations were significantly reduced after subjects received SPC treatment compared with the effects of placebo treatment. The time span of serums IL-17A, IL-10, and TNF-𝛼 restored to a normal level was shorter in the SPC group than the placebo group. Moreover, SPC could decrease the level of serum miR-155 and upregulated miR-21 expression. However, heart function and serum SOD and MDA levels did not significantly differ between the SPC and the placebo groups. Taken together, these data demonstrate that SPCs may effectively expedite the process of recovering from HADAS.

The mechanism underlying the accelerated recuperation of HADAS subjects in the SPC group remains unclear. Although HADAS occurs at lower altitudes after subjects return from high altitudes, the occurrence and progression of HADAS involve damage from hypoxia and reoxygenation [21]. The subjects suffered from hypoxia while living at high altitude, but upon returning to a lower altitude, they were suddenly exposed to the oxygen content of the lowlands, approximately 21%. As reoxygenation proceeded, the O_2_ percentage increased by 6–8% [1]. Hypoxia and reoxygenation injury can induce oxidative stress-related homeostatic dysregulation [26, 27] and other related physiological and pathological changes, which in turn can activate many signaling pathways in neurons, myocardial cells, and endothelial cells. Several studies have reported that hypoxia and reoxygenation cause injury and inflammatory response to the nervous system, myocardium, and small intestine, as well as arthrosis [28–35], the appearance of painful symptoms, and increasing levels of myocardial enzymes.

SPCs are a patented Chinese medicine made of raw materials that include flavonoids, quercetin, kaempferol, isorhamnetin, astragaloside, and atractylenolide III [16]. SPC ingredients can play important roles as antioxidants, which prevent oxidative stress-induced damage, inflammation, and apoptosis [36–41]. Our previous study found that serums IL-17A and TNF-*α* were elevated and the IL-10 level was descended upon the individuals return to sea level from a high-altitude region, and the level of serums IL-10 and TNF-*α* returns to normal at 50^th^ day or even earlier, as well as the IL-17 level restored to normal almost needs 100 days [21]. Herein, we demonstrated that SPC played an important role to accelerate inflammatory mediator return to a normal level, suggesting SPC might have an anti-inflammation effect due to its contained variety of anti-inflammatory ingredients. Additionally, serum MDA and SOD levels have no difference between the SPC and the placebo groups. Our pervious study showed that the serum of MDA and SOD in HADAS subjects had already return to normal levels at 50^th^ day from high altitude to sea level [21]. Therefore, we speculated that these data could not mean the antioxidant effect of SPC was not working and the antioxidant effect of SPC needs to clarify in further study.

It is well known that the abnormal expression and location of miRNA are involved in several pathophysiology and disease, including inflammatory response, ischemia reperfusion injury, and cancer, through regulated target gene posttranscription [42]. Several documents showed that miR-155 promoted inflammatory response involved in atherosclerosis [43], obesity-induced renal inflammation [44, 45], and ischemia-reperfusion injury [46–48]. In this study, we found that the serum miR-155 level was higher at baseline than at 50^th^ day and 100^th^ day. Given the above results about inflammatory mediator, we speculated that miR-155 was involved in inflammatory response in HADAS. Moreover, SPC decreased the level of serum miR-155, suggesting that SPC suppresses inflammatory response in HADAS through downregulation of miR-155 expression. miR-21 plays an import role to attenuate hypoxia-reoxygenation injury [49–52]. Our previous study proved that hypoxia-reoxygenation injury is involved in HADAS [21]. In this study, the level of serum miR-21 was lower at baseline than at 50^th^ day and 100^th^ day for both groups. After subjects received SPC treatment, the serum miR-21 level increased rapidly compared to the placebo group, hinting that miR-21 is involved in HADAS, and SPC might elevate miR-21 to attenuate hypoxia-reoxygenation injury.

Our trial has some limitations. First, all of the subjects were young men, and the majority was between 18 and 35 years old. Second, the spans between evaluation time points were too long to evaluate certain parameters, such as CK and IL-17A, which had already recovered to similar, normal levels in both groups before measurements were made. Most subjects were particularly reluctant to have blood drawn and only agreed to have their symptoms evaluated and biological measurements taken at day 3 and weeks 7 and 14. All of these factors may have introduced bias into the results.

## 5. Conclusions

In this trial, the oral administration of SPCs to subjects with mild or moderate HADAS significantly decreased HADAS scores in the trial group compared with effects in the placebo group at week 14. SPC treatment improves hypoxia-reoxygenation cause injury and inflammatory response and promotes subjects' body return to normal state. These findings from this multicenter trial suggest that oral SPCs may improve HADAS symptoms and expedite recovery via improve hypoxia-reoxygenation cause injury and inflammatory response.

## Figures and Tables

**Table 1:** Baseline characteristics of subjects at randomization according to the study group.

	SPC group (*n* = 146)	Placebo group (*n* = 142)	*p* value
*Demographic characteristics*			
Chongqing (180 m) (%)	44 (30.13%)	42 (29.58%)	0.92
Kunming (1800 m) (%)	34 (23.29%)	33 (23.24%)	0.99
Zhengzhou (110 m) (%)	38 (26.03%)	37 (26.06%)	0.95
Wuwei (1500 m) (%)	30 (20.55%)	30 (21.12%)	0.90
Age, years (SD, range)	25.09 (5.08, 18-35)	24.60 (4.66, 18-34)	0.40
Race, Han (%)	146 (100%)	142 (100%)	1.00
Sex, male (%)	146 (100%)	142 (100%)	1.00
*Work intensity*			
Mild	42 (28.77%)	44 (30.98%)	0.42
Moderate	86 (58.90%)	88 (61.97%)	0.63
Severe	14 (9.58)	14 (9.85%)	0.94
*Smoking*	114 (78.08%)	106 (74.64%)	0.49
*Alcohol consumption*	113 (77.39%)	109 (76.76%)	0.90
*AMS occurrence*	19 (13.01%)	17 (11.97%)	0.79

The data are presented as *n* (%) or the mean ((SD; range) or (SE, 95% CI)). AMS occurrence: subjects suffered from acute mountain sickness (AMS) at high altitude. Statistical differences between groups were tested for using a *t* test for quantitative data and a chi-square test for categorical data.

**Table 2:** Primary outcomes for research worker-rated HADAS scores.

	Placebo group (*n* = 146)	SPC group (*n* = 142)	Mean difference from placebo^†^	*p*_*m*_ value^∗^	*p*_*t*_ value^‡^	*p*_*g*_ value^§^	*p*_*g*+*t*_ value^||^
Baseline	12.80 (0.28, 12.25-13.34)	13.08 (0.28, 12.52-13.63)					
Week 7	5.26 (0.16, 4.96 - 5.59)	3.95 (0.16, 3.64 - 4.26)	-1.34 (0.20, -1.74- -0.95)	<0.001			
Week 14	2.80 (0.12, 2.57 - 3.04)	1.36 (0.12, 1.13 - 1.58)	1.47 (0.20, -1.87- -1.07)	<0.001			
Collapsed across time, baseline to week 14					<0.001	<0.001	<0.001

^†^Values are the adjusted mean change (SE, 95% CI). ^∗^Comparisons of the mean differences were made at weeks 7 and 14 from the final adjusted linear mixed model, p_m_. ^‡^The overall change over time, *p*_*t*_. ^§^The average group difference, *p*_*g*_. ^||^The interaction between time and group, *p*_*t*+*g*_.

**Table 3:** Analysis of symptom incidences of HADAS.

Symptoms	Baseline			Week 7				Week 14			
	Placebo group (*n* = 142)	SPC group (*n* = 146)	*p* value	Placebo group (*n* = 142)	SPC group (*n* = 146)	RR	*p* value	Placebo group (*n* = 142)	SPC group (*n* = 146)	RR	*p* value
Sleepiness	97 (68.30%)	87 (59.58%)	0.13	50 (35.21%)	35 (23.97%)	0.58	0.04	23 (16.20%)	9 (6.16%)	0.34	0.009
Insomnia	60 (42.25%)	62 (42.46%)	0.94	44 (30.99%)	26 (17.81%)	0.51	0.02	26 (18.31%)	10 (10.96%)	0.44	0.03
Unresponsiveness	93 (65.49%)	100 (68.49%)	0.63	60 (42.25%)	44 (30.13%)	0.58	0.03	29 (20.42%)	14 (9.59%)	0.47	0.04
Memory loss	86 (60.56%)	88 (60.27%)	0.99	59 (41.54%)	41 (28.08%)	0.55	0.02	28 (19.72%)	17 (11.64%)	0.70	0.28
Agitation	52 (36.61%)	45 (30.82%)	0.27	27 (19.01%)	15 (10.27%)	0.49	0.04	15 (10.56%)	10 (6.85%)	0.58	0.23
Headache	66 (46.47%)	67 (45.89%)	0.97	44 (33.10%)	32 (21.91%)	0.58	0.04	23 (16.20%)	12 (8.22%)	0.46	0.04
Dizziness	100 (70.42%)	111 (79.02%)	0.28	67 (47.18%)	47 (32.19%)	0.53	0.01	44 (30.99%)	18 (12.33%)	0.42	0.004
Throat pain or discomfort	50 (35.21%)	43 (29.45%)	0.26	24 (16.90%)	25 (17.12%)	1.06	0.87	12 (8.45%)	13 (8.90%)	1.40	0.41
Coughing	97 (68.30%)	114 (78.08%)	0.06	57 (40.14%)	40 (27.39%)	0.54	0.02	31 (21.83%)	11 (7.53%)	0.42	0.01
Expectoration	68 (47.88%)	68 (46.57%)	0.75	28 (19.72%)	27 (18.49%)	0.90	0.74	19 (13.38%)	6 (4.11%)	0.38	0.03
Chest tightness	64 (45.07%)	55 (37.67%)	0.18	41 (28.87%)	24 (16.43%)	0.47	0.01	17 (11.97%)	7 (4.79%)	0.37	0.03
Flustering	82 (57.74%)	74 (50.68%)	0.25	52 (36.61%)	36 (24.66%)	0.57	0.03	28 (19.72%)	13 (8.90%)	0.47	0.02
Increased appetite	68 (47.88%)	67 (45.89%)	0.77	49 (34.51%)	35 (21.91%)	0.60	0.05	22 (15.49%)	9 (6.16%)	0.51	0.08
Decreased appetite	58 (40.84%)	56 (38.35%)	0.64	44 (30.99%)	32 (21.92%)	0.60	0.06	21 (14.79%)	8 (5.48%)	0.32	0.009
Diarrhea	66 (46.47%)	50 (36.98%)	0.10	22 (15.49%)	25 (17.12%)	1.12	0.73	8 (5.63%)	0 (0.00%)	0.10	0.03
Abdominal distention	48 (33.80%)	50 (34.24%)	0.97	37 (26.06%)	23 (15.75%)	0.97	0.99	12 (8.45%)	8 (5.48%)	0.71	0.46
Abdominal pain	37 (26.05%)	35 (23.97%)	0.71	19 (13.38%)	14 (9.59%)	1.11	0.71	5 (3.52%)	1 (0.07%)	0.17	0.12
Lumbago	50 (35.21%)	42 (28.76%)	0.23	23 (16.19%)	24 (16.43%)	1.37	0.23	17 (11.97%)	14 (11.64%)	0.77	0.51
Arthralgia	68 (47.88%)	61 (41.78%)	0.35	25 (17.61%)	24 (16.44%)	1.25	0.35	19 (13.38%)	11 (7.53%)	0.56	0.14

The data are presented as the number (%) of subjects. RR represents the relative risk for the SPC versus the placebo group. The symptom incidences were compared using Fisher's exact tests.

**Table 4:** Analysis of the overall proportions of HADAS severity in subjects.

HADAS severity	Week 7				Week 14			
	Placebo group (*n* = 142)	SPC group (*n* = 146)	RR	*p* value	Placebo group (*n* = 142)	SPC group (*n* = 146)	RR	*p* value
Moderate reaction (*n*, %)	0 (0.00%)	0 (0.00%)			0 (0.00%)	0 (0.00%)		
Mild reaction (*n*, %)	62 (43.66%)	34 (23.29%)	0.17	<0.001	13 (9.15%)	1 (0.68%)	0.04	0.004
Almost no reaction (*n*, %)	80 (56.34%)	112 (76.71%)			129 (90.85%)	145 (99.32%)		

The data are presented as the number (%) of subjects. RR represents the relative risk for the SPC versus the placebo group. Symptom severities were compared using Fisher's exact tests.

**Table 5:** Analysis of secondary outcomes.

Secondary outcome	Baseline		Week 7		Week 14		*p*_*t*_ value^‡^	*p*_*g*_ value^§^	*p*_*g*+*t*_ value^||^
	Placebo group	SPC group	Placebo group	SPC group	Placebo group	SPC group			
*Myocardial enzymes*									
CK (IU/L)	122.14 (2.94, 116.42- 128.06)	124.14 (3.34, 117.52- 130.75)	116.53 (2.12, 112.33 - 120.73)	92.30 (1.92, 88.51- 96.10)^∗^	104.90 (2.64, 99.67 - 110.13)	99.60 (2.25, 95.13 - 104.08)	<0.001	<0.001	<0.001
CK-MB (IU/L)	21.70 (0.61, 20.49- 22.91)	21.37 (0.49, 20.38-22.35)	19.42 (0.24, 18.95- 19.90)	15.47 (0.23, 14.99- 15.95)^∗^	17.48 (0.32, 16.84 - 18.13)	15.89 (0.16, 15.56 - 16.21)^∗^	<0.001	<0.001	<0.001
LDH (IU/L)	198.89 (2.29, 194.37- 203.41)	195.87 (2.80, 189.43- 199.48)	170.53 (3.15, 169.37 - 183.31)	156.95 (2.70, 143.89 - 155.19)^∗^	151.03 (2.65, 145.78 - 156.27)	133.47 (2.01, 129.49 - 137.46)^∗^	<0.001	<0.001	<0.001
*Heart function*									
HR (beats/min)	75.07 (0.45, 74.17- 75.96)	74.36 (0.95, 72.47- 76.25)	71.14 (0.45, 70.24 - 72.03)	70.89 (0.67, 69.56 - 72.22)	70.03 (0.67, 68.69 - 71.37)	70.56 (0.49, 69.56 - 71.50)	<0.001	0.84	0.59
LVEF (%)	64.22 (0.46, 63.30- 65.14)	64.07 (0.40, 63.27- 64.87)	63.51 (0.39, 62.72 - 64.30)	63.45 (0.47, 62.62 - 64.38)	58.87 (0.35, 58.16 - 59.58)	57.90 (0.34, 57.22 to 58.58)	<0.001	0.26	0.405
LVFS (%)	34.12 (0.38, 33.36- 34.88)	34.63 (0.30, 34.03- 35.24)	34.82 (0.27, 34.27 - 35.37)	34.87 (0.31, 34.26 - 35.49)	31.45 (0.29, 30.88 - 32.03)	30.92 (0.22, 30.48 to 31.36)	<0.001	0.94	0.18
*Pulmonary artery inner diameter (mm)*	27.34 (0.12, 24.38- 30.30)	26.84 (0.09, 24.62- 29.05)	24.14 (0.07, 24.00 - 24.28)	24.27 (0.05, 24.16 - 24.37)	20.83 (0.12, 20.58 - 21.08)	20.93 (0.05, 20.83 to 21.03)	<0.001	0.11	0.367
PAOV (m/s)	81.78 (0.54, 80.7- 82.86)	80.67 (0.47, 79.73- 81.60)	95.14 (0.52, 94.11 - 96.17)	93.26 (0.54, 92.19 - 94.34)	81.78 (0.54, 80.70 - 82.86)	81.01 (0.51, 79.99 to 82.02)	<0.001	0.01	0.60
PASP (mmHg)	2.79 (0.31, 2.7- 2.85)	2.73 (0.30, 2.67- 2.79)	3.64 (0.07, 3.51 - 3.77)^∗^	3.45 (0.35, 3.38 - 3.52)	2.73 (0.03, 2.67 - 2.79)	2.71 (0.03, 2.64 to 2.78)	<0.001	0.18	0.01

The data are presented as the means (SE, 95% CI). RBC = red blood cells, Hb = hemoglobin, Hct = hematocrit, MCV = mean corpuscular volume. CK = creatine kinase, CK-MB = creatine kinase-MB, LDH = lactate dehydrogenase. HR = heart rate, LVEF = left ventricular ejection fraction, LVFS = left ventricular fractional shortening, PAOV = pulmonary artery opening velocity, PASP = pulmonary artery systolic pressure. ^∗^Comparisons of the mean differences were made at weeks 7 and 14 from the final adjusted linear mixed model, *p* < 0.05. ^‡^The overall change over time, *p*_*t*_. ^§^The average group difference, *p*_*g*_. ^||^The interaction between time and group, *p*_*t*+*g*_.

## Data Availability

The data used to support the findings of this study are available from the corresponding author upon request.

## Supplementary Materials

Supplementary Materials This supplement file included the CONSORT 2010 table, diagnostic and scoring criteria for fixed period high-altitude deacclimatization syndrome, method of biological measurements and cardiac function, and sample size calculation.
