# Supplementary material for: Efficacy of Shenqi Pollen Capsules for High-Altitude Deacclimatization Syndrome via Suppression of the Reoxygenation Injury and Inflammatory Response
Source: J Immunol Res. 2019 Nov 15;2019:4521231. doi: 10.1155/2019/4521231 (PMC6881745; doi:10.1155/2019/4521231)
Supplement: Supplementary Materials — This supplement file included the CONSORT 2010 table, diagnostic and scoring criteria for fixed period high-altitude deacclimatization syndrome, method of biological measurements and cardiac function, and sample size calculation. [file 4521231.f1.doc]

**Efficacy and safety of Shenqi pollen capsules for high altitude de-acclimatization syndrome: A Randomized Controlled Trial**

Binfeng He^1^*, Mingdong Hu^1^*, Zhihui Liang^2^, Qianli Ma^1^, Yunhai Zi^1^, Zhiwei Dong^3^, Qi Li^1^, Yongjun Luo^4^, Guisheng Qian^1^, Liang Guo^1^, Kexiong Lin^5^, Zhenyu Liu ^6§^, & Guansong Wang^1§^

^1^Institute of Respiratory Diseases, Xinqiao Hospital of the Third Military Medical University, Chongqing 400037, China

^2^Bethune International Peace Hospital of PLA, Shijiazhuang, Hebei 050000, China

^3^Center for Disease Control and Prevention of Zhengzhou City, and First Affiliated Hospital of Zhengzhou University, Zhengzhou, Henan 450000, China

^4^College of High Altitude Military Medicine, Third Military Medical University, Chongqing, 400038, China

^5^Department of Respiration, Tongren City People’s Hospital, Guizhou 554300, China

^6^Department of Emergency Medicine, the First Hospital of Nanchang University, Nanchang 330006, China

§Correspondence:

wanggs2003@hotmail.com, wanggs@tmmu.edu.cn, 3123891447@qq.com

Tel.: +86 23 6875 5644, Fax: +86 23 6521 1653

*These authors contributed equally to this work


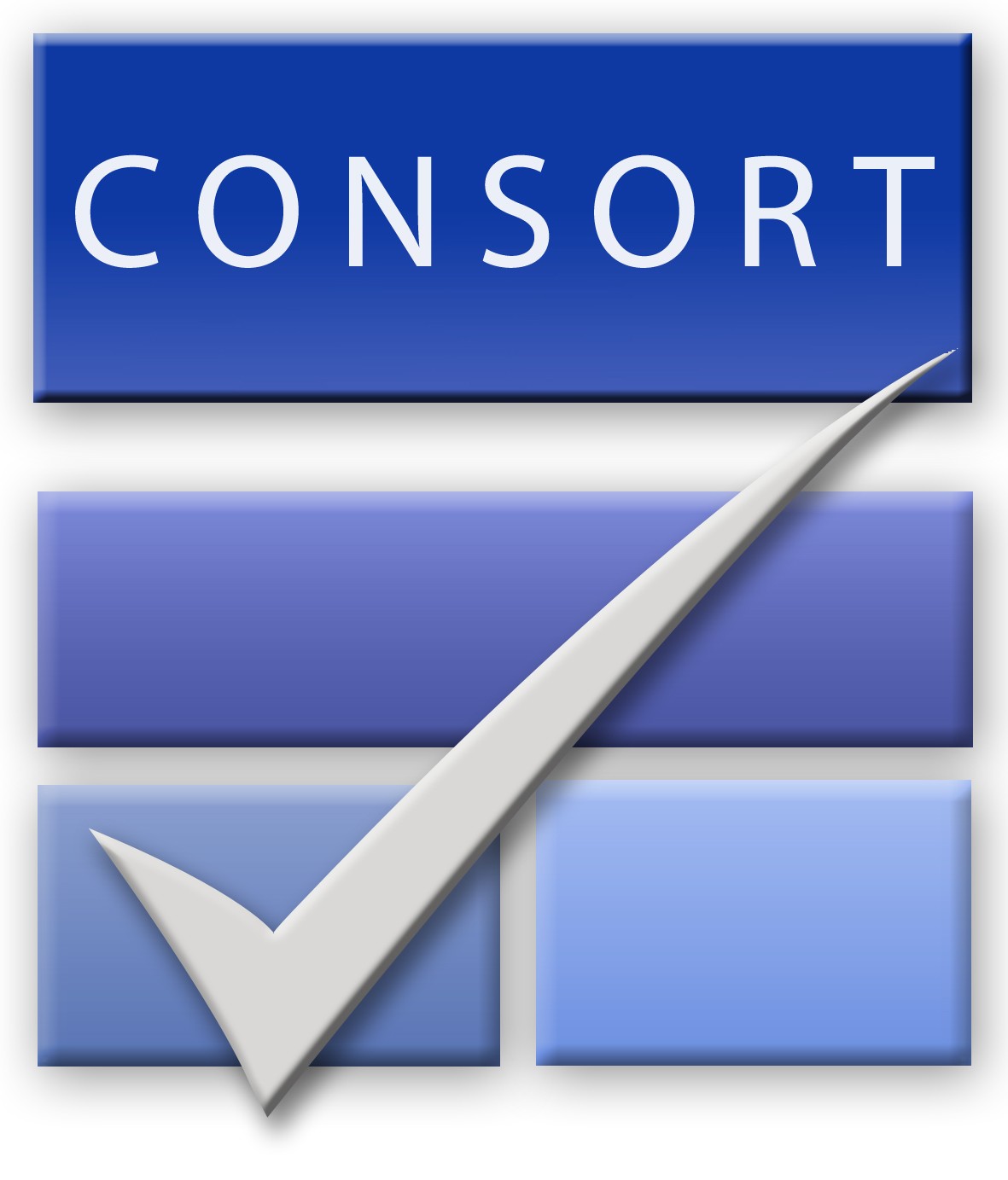
CONSORT 2010 checklist of information to include when reporting a randomised trial*

| Section/Topic | Item No | Checklist item | Reported on page No |
| --- | --- | --- | --- |
| Title and abstract | | | |
|  | 1a | Identification as a randomised trial in the title | 1 |
|  | 1b | Structured summary of trial design, methods, results, and conclusions (for specific guidance see CONSORT for abstracts) | 2 |
| Introduction | | | |
| Background and objectives | 2a | Scientific background and explanation of rationale | 3-6 |
|  | 2b | Specific objectives or hypotheses | 6 |
| Methods | | | |
| Trial design | 3a | Description of trial design (such as parallel, factorial) including allocation ratio | 6-8 |
|  | 3b | Important changes to methods after trial commencement (such as eligibility criteria), with reasons | N/A |
| Participants | 4a | Eligibility criteria for participants | 7 |
|  | 4b | Settings and locations where the data were collected | 6 |
| Interventions | 5 | The interventions for each group with sufficient details to allow replication, including how and when they were actually administered | 9-10 |
| Outcomes | 6a | Completely defined pre-specified primary and secondary outcome measures, including how and when they were assessed | 13 |
|  | 6b | Any changes to trial outcomes after the trial commenced, with reasons | N/A |
| Sample size | 7a | How sample size was determined | 14 |
|  | 7b | When applicable, explanation of any interim analyses and stopping guidelines | 14 |
| Randomisation: |  |  |  |
| Sequence generation | 8a | Method used to generate the random allocation sequence | 8 |
|  | 8b | Type of randomisation; details of any restriction (such as blocking and block size) | 8 |
| Allocation concealment mechanism | 9 | Mechanism used to implement the random allocation sequence (such as sequentially numbered containers), describing any steps taken to conceal the sequence until interventions were assigned | 8 |
| Implementation | 10 | Who generated the random allocation sequence, who enrolled participants, and who assigned participants to interventions | 8 |
| Blinding | 11a | If done, who was blinded after assignment to interventions (for example, participants, care providers, those assessing outcomes) and how | 8 |
|  | 11b | If relevant, description of the similarity of interventions | 8-9 |
| Statistical methods | 12a | Statistical methods used to compare groups for primary and secondary outcomes | 14-15 |
|  | 12b | Methods for additional analyses, such as subgroup analyses and adjusted analyses | 14-15 |
| Results | | | |
| Participant flow (a diagram is strongly recommended) | 13a | For each group, the numbers of participants who were randomly assigned, received intended treatment, and were analysed for the primary outcome | 15 |
|  | 13b | For each group, losses and exclusions after randomisation, together with reasons | Figure1 |
| Recruitment | 14a | Dates defining the periods of recruitment and follow-up | 15-16 |
|  | 14b | Why the trial ended or was stopped | N/A |
| Baseline data | 15 | A table showing baseline demographic and clinical characteristics for each group | Table1 |
| Numbers analysed | 16 | For each group, number of participants (denominator) included in each analysis and whether the analysis was by original assigned groups | 15-16 |
| Outcomes and estimation | 17a | For each primary and secondary outcome, results for each group, and the estimated effect size and its precision (such as 95% confidence interval) | 15-17 |
|  | 17b | For binary outcomes, presentation of both absolute and relative effect sizes is recommended | 15-17 |
| Ancillary analyses | 18 | Results of any other analyses performed, including subgroup analyses and adjusted analyses, distinguishing pre-specified from exploratory | 15-17 |
| Harms | 19 | All important harms or unintended effects in each group (for specific guidance see CONSORT for harms) | N/A |
| Discussion | | | |
| Limitations | 20 | Trial limitations, addressing sources of potential bias, imprecision, and, if relevant, multiplicity of analyses | 21 |
| Generalisability | 21 | Generalisability (external validity, applicability) of the trial findings | 21 |
| Interpretation | 22 | Interpretation consistent with results, balancing benefits and harms, and considering other relevant evidence | 19-20 |
| Other information | | |  |
| Registration | 23 | Registration number and name of trial registry | 7 |
| Protocol | 24 | Where the full trial protocol can be accessed, if available | 7 |
| Funding | 25 | Sources of funding and other support (such as supply of drugs), role of funders | 22 |

*We strongly recommend reading this statement in conjunction with the CONSORT 2010 Explanation and Elaboration for important clarifications on all the items. If relevant, we also recommend reading CONSORT extensions for cluster randomised trials, non-inferiority and equivalence trials, non-pharmacological treatments, herbal interventions, and pragmatic trials. Additional extensions are forthcoming: for those and for up to date references relevant to this checklist, see [www.consort-statement.org](http://www.consort-statement.org/)

**Diagnostic and scoring criteria for fixed period high-altitude de-acclimatization syndrome**

The diagnostic and scoring criteria for high altitude de-acclimatization syndrome are based on epidemiological study data.

1. Essential diagnostic criteria for high altitude de-acclimatization syndrome

(1) Adult ≤ 60 years old. (2) Recent return to lower altitude from a higher altitude. (3) Three or more of the following symptoms: fatigue, sleepiness, insomnia, unresponsiveness, memory loss, fidgety, headache, throat pain or discomfort, coughing, expectoration, chest tightness, flustering, increased appetite, decreased appetite, expectoration, diarrhea, abdominal distention, abdominal pain, lumbago, or arthralgia. (4) No significant relief of symptoms after 3 days of simple medication administered after the return to lower altitudes.

2. Auxiliary diagnostic criteria for high altitude de-acclimatization syndrome

(1) Blood routine: RBC, Hb, and Hct levels high above baseline. (2) Myocardial enzymes: CK-MB and LDH levels above those of individual’s native to low altitudes. (3) Urine: Microalbuminuria above that of individual’s native to low altitudes. (4) Heart function: Pulmonary arterial pressure slightly higher than that of individuals native to high altitudes accompanied by left or right ventricular systolic and diastolic dysfunction. Raised Tei index coupled with low values of left ventricular ejection fraction (LVEF), right ventricular ejection fraction (RVEF), left ventricular fractional shortening (LVFS), and right ventricular fractional shortening (RVFS). (5) Brain function: Dysfunction of immediate short-term memory. (6) Hepatic function: Total bilirubin, ALT, and AST levels higher than those of individual’s native to low altitudes.

Diagnosis of high altitude de-acclimatization included essential conditions and one Auxiliary condition.

3. Exclusion criteria

(1) Symptoms directly attributable to primary diseases affecting the cardiovascular, respiratory, nervous, urinary, and hematological systems. (2) Cancer or leukemia. (3) Any history of highland heart disease or high-altitude polycythemia. (4) Recent history of flu, upper respiratory tract infection, infectious diarrhea, or similar symptoms.

4. Classification and scoring criteria of the symptoms of high-altitude de- acclimatization syndrome (Tables 1 and 2).

## Table 1. Classification and scoring criteria of the symptoms of high-altitude de-acclimatization syndrome

|  | | |
| --- | --- | --- |
| Classification | Judgment standards | Scoring standards |
| ± | Mild symptoms with no impact on daily life | 0 |
| + | Mild symptoms with slight impact on daily life, ameliorated after drug regimen | 1 |
| ++ | Severe symptoms affect daily life, somewhat alleviated after drug regimen | 2 |
| +++ | Severe symptoms affect daily life, no significant relief after drug regimen | 3 |

## Table 2. Grading of high-altitude altitude de-acclimatization syndrome

|  | |
| --- | --- |
| Classification | Diagnostic criteria |
| Almost no reaction (±) | Suspected symptoms (±) or 0–5 total points |
| Mild reaction (+) | Slight symptoms (+) or 6–15 total points |
| Moderate reaction (++) | More serious symptoms (++) or 16–25 total points |
| Severe reaction (+++) | Very serious symptoms (+++) or 26 or more total points |

5. Symptom scores

Symptom scores were evaluated according to the scoring criteria for fixed-duration high-altitude de-acclimatization syndrome.

**Biological measurements**

1. Collection and analysis of blood samples

Morning fasting venous blood (1 ml) was collected (with EDTA-K2), and samples were assayed within 2 hours using an hematology analyzer (Sysmex XE - 2100, Japan) at the Xinqiao Hospital in Chongqing, the 478th Hospital in Kunming, First Hospital of Zhengzhou University in Zhengzhou and Wuwei City People’s Hospital in Wuwei. The operation was performed in strict according with the standard SOP. All equipment was used in accordance with the manufacturers’ protocols. The same types of equipment were used at all three sites and they showed statistically similar levels of efficiency (P <0.01).

2. Measurement of myocardial enzymes

Morning fasting venous blood (3 ml) was collected, centrifuged at 4000 r/min for 10 min to separate serum, and stored at -80°C before assay. The concentrations of CK, CK-MB, and LDH were measured at Xinqiao Hospital on a fully automated biochemistry analyzer (Olympus Au2700, Japan).

**Measurement of cardiac function**

Color Doppler ultrasound system (GE LOGIQ-3) was used to measure Heart rate (HR), Left ventricular ejection fraction (LVEF), Left ventricular fractional shortening (LVFS), Pulmonary artery opening velocity (PAOV) and Pulmonary artery systolic pressure (PASP). The heart function of all subjects was measured by anultrasonogra physpecialist from Xinqiao Hospital.

**Smple size calculation**

According to the data of our previous study, the assumed mean differences at week 7 and week 14 were 0.8 and 0.84, respectively, between the two treatment groups. The standard deviations (SDs) of the placebo group’s reduction in HADAS score at weeks 7 and 14 were 1.7 and 2, respectively, and the corresponding SDs of the SPC group was 2 and 2.5, respectively. The power was 0.80, and α was 0.05. This trial allowed for a 20% loss to follow-up. As the subjects lived and worked together in a unit, all of the subjects with mild or moderate HADAS within a unit were considered as a cluster and enrolled in our trial. This resulted in a total number of subjects of 288.

**Table 3.** The distribution of subjects in the trial

| Center | SPC groups | | | Placebo group | | |
| --- | --- | --- | --- | --- | --- | --- |
|  | unit 1 | unit 2 | unit 3 | unit 4 | unit 5 | unit 6 |
| Chongqing | 17 | 10 | 17 | 16 | 14 | 12 |
| Kunming | 17 | 17 |  | 17 | 16 |  |
| Zhengzhou | 15 | 13 | 10 | 10 | 12 | 15 |
| Wuwei | 14 | 16 |  | 13 | 17 |  |
